# Turning by External Manipulation of the Abdomen

**Published:** 1855-11

**Authors:** 


Turning by External Manipulation of the Abdomen.—M. Mattei, a Corsican Professor of Midwifery, draws attention to the feasibility of this procedure in rectifying the position of the child. By its means, he says he has frequently, in pelvic presentation, been enabled to raise the pelvis, and press down the head into its proper position, repeated attempts to do this causing no ill consequences. The diagnosis of the position of the child by palpation of the abdomen and auscultation is easy, and the performance of the operation at the latter end of pregnancy is not difficult, if attempted before the child is engaged within the pelvis. During the eighth, or at the commencement of the ninth month, is the preferable period.—(*Gazette Médicale.* No. 23.)

[This mode of turning has been frequently resorted to in Germany.]—*Ibid.*

